# Causal thinking and causal language in epidemiology: a cause by any other name is still a cause: *response to Lipton and Ødegaard*

**DOI:** 10.1186/1742-5573-3-7

**Published:** 2006-06-06

**Authors:** Clarence C Tam

**Affiliations:** 1 Infectious Disease Epidemiology Unit, Department of Infectious & Tropical Diseases, London School of Hygiene & Tropical Medicine, Keppel Street, London WC1E 7HT, UK

## 

I have great sympathy with the thoughts of Lipton and Ødegaard [1] – the assessment and communication of "causal" associations is a source of continual frustration for epidemiologists. The authors' lucid account of the use of causal language in epidemiology can essentially (if rather unflatteringly) be simplified to the following: it is impossible to prove that *X *causes *Y*; the statement *"Smoking causes lung cancer" *is thus no more informative than the statement *"Smoking two packs a day for *N *years increases your risk of lung cancer ten-fold"*. In fact, it is less informative and even misleading. The authors argue that such causal statements are redundant, logically indefensible and should be avoided in favour of more detailed descriptions of the process by which such associations are established (the "story", as the authors put it). The latter are, in themselves, sufficient causal statements (the notion of "letting the data speak for themselves") and nothing is gained by making subjective attributions of causality.

It is, of course, difficult to argue with such reasoning. Sensible epidemiologists would shy away from stating that a particular *X *causes a particular *Y*, because they know that, in purely statistical terms, there is always a possibility that they could be wrong. There is, in fact, much with which I agree in Lipton and Ødegaard's paper. To their call for "telling a good story", which they argue lies "not in the naming of something as causal, but in the actual rigor of the analysis" [1] (p8), I would add that it is not just rigour in the analysis that is required, but also rigour in asking good questions involving testable hypotheses, rigour in study design and execution, and rigour in the description of results. There are, however, notable points on which I disagree. The authors begin with a short caveat, claiming not to attempt "to revisit the long-standing debate between realism and pragmatism in science". I find this somewhat strange as, to me, this issue is at the crux of the argument. The common description of epidemiology as a "pragmatic" science is rather unfortunate. The term "pragmatic" suggests some form of compromise between objective "realism" and the vicissitudes of everyday life (see Appendix Footnote 1). This is somewhat ironic; I see no compromise in epidemiology as a field that applies scientific methods, however imperfect, to study everyday problems with the ultimate aim of improving the health of individuals and populations. Acceptance of its imperfections is, on the other hand, a very "realist" attitude. In a very "pragmatic" sense then, the epidemiologist can say that, having assessed the available evidence, smoking is a cause of lung cancer. The philosopher, however (and I say this meaning no disrespect to the field), can say nothing about any causal link between smoking and lung cancer – either in the "pragmatic" or objective senses – other than the fact that they can say nothing about any causal link between smoking and lung cancer (and, indeed, generally). Lipton and Ødegaard do seem to accept this "pragmatic" role of epidemiology, as they assert that they are interested in "the ability to manipulate the world, to predict and intervene" [1] (p3), but do not feel that this is relevant to their discussion of causation in epidemiology.

My main difficulty with Lipton and Ødegaard's position perhaps stems from my love of the written language. The ability to string words together on a piece of paper to inspire a sense of knowledge, excitement, wonder, sadness or depression in someone perhaps sitting thousands of miles away whom you may never have met is without doubt one of the most honourable and worthwhile of human activities. A whisper in someone's ear, a casual conversation, a speech at a convention are all eventually lost to dispersing airwaves or adulterated by time and memory. A well-constructed written sentence is, at least in principle, timeless.

It is from this rather romantic perspective that I find myself pained by the awkwardness with which epidemiologists must describe associations between exposures and health outcomes. The term "cause" is steeped in great history and philosophy; it is used to describe an objective ideal by which one event invariably leads to another, and as such is often thought to have limited use in our everyday world.

Imagine the Aristotelian notions of an "ideal" universe, made up of perfect geometrical bodies, and the observable "real" universe, an imperfect approximation of the ideal in which, for example, spherical bodies appear to be spheres, but upon closer examination are shown, by nature of their imperfection, to be made up of flat surfaces. This (admittedly somewhat naïve) scenario provides us with a visual analogy of how epidemiologists might view the world. There is, we suspect, an ideal, true, but unobservable "causal" association between *X *and *Y*. As with other scientific fields, we aim to model this ideal universe using our imperfect methods and, from our ensuing observations, make some sort of inference about the true causal association. The question then becomes how closely our imperfect models resemble the ideal universe they are intended to reflect – if you like, how small the area of those flat surfaces that make up those spherical bodies is. The smaller the surfaces, the smaller the error in our observations, and the closer our imperfect geometrical representations will resemble perfect spherical bodies.

Lipton and Ødegaard argue that the concept of causation in the ideal universe, by nature of its being unobservable, is irrelevant. One might even wonder whether causation even exists at all in the ideal universe; maybe things just "happen". Consider two identical twins who share in common not just their genetic make-up, but everything else in their lives. They live in the same house, eat exactly the same foods, have the same jobs, think the same thoughts at the same time; in fact, at any given point in time, the two are completely interchangeable. This is perhaps the closest we could ever get to a real interpretation of our ideal universe. Suppose both twins take up smoking two packs of cigarettes a day at the age of 20, which they smoke at exactly the same times. Both then go on to develop lung cancer at the age of 50. The fact that they have been smoking two packs of cigarettes every day for the past 30 years would give us no information as to whether their lung cancer was caused by smoking, in much the same way as their age, sex, occupation, genetic make-up or any of the other factors that the twins share in common (which is everything) would tell us nothing about the cause of their lung cancer. Suppose, however, that only one of the twins had taken up smoking. Only the smoker goes on to develop lung cancer at the age of 50 (I ignore the possible effects of passive smoking here). I imagine that most people would be prepared to bet good money on smoking being the cause of the smoker's cancer in this case, since it is the only factor that distinguishes the twins. But what if both twins had taken up smoking and only one subsequently developed lung cancer? Again, we would be in a quagmire as to what to say about smoking as a cause of lung cancer. We might venture to say that, all else being equal, smoking induces cancer with a certain probability. Of course, in the ideal universe nothing is ever equal, for that would require two instances of the same type of event occurring in the same place at the same time, and all the preceding events for the two events to have occurred in exactly the same point in space-time, apart from the one that was subsequently termed the "cause". Asserting that this one cause effects the outcome with a certain probability would be tantamount to saying that God does indeed play dice, a question perhaps best left to quantum physicists. I imagine, however, that most epidemiologists would not be prepared to accept such an interpretation. After all, our observations tell us that things do not occur at random; health outcomes cluster among individuals with certain characteristics, and it seems unreasonable to suggest that if smoking were not a true cause of lung cancer, we should repeatedly observe this association simply by chance. Our idea of causation is based on a belief that we can assign individuals into groups with certain common, relevant characteristics, disregarding factors that we judge to be uninformative about a given association and, therefore, ignorable. Otherwise, the notion of causation does not make sense. That we, as epidemiologists, have this belief attests to its being relevant and important, regardless of whether it is unobservable.

It is here that I find a frank contradiction in Lipton and Ødegaard's argument. They agree that the concept of causation is important in epidemiology, and even agree that terms commonly used to imply causal relationships, such as "X *increases the risk of *Y *by*" can be used in much the same sense as "X *causes *Y" [1] (p5). The authors do not seem to be objecting to the use of the term "cause" on the grounds it describes something that is qualitatively different. It is thus hard to see what they find so disagreeable about its use. Perhaps they simply do not like the word "cause", preferring instead more descriptive associational statements that they nevertheless seem to agree amount to qualitatively the same thing.

Lipton and Ødegaard's assertion that statements of association between exposures and outcomes are in themselves sufficient causal statements is based on the strong assumption that, for any two given associational statements, the degree of evidence is equivalent – that the flat surfaces making up those imperfect spheres are of equal area. I doubt, however, whether most epidemiologists would lend equal weight to any pair of causal statements, such as *"Smoking *X *packs a day for *N *years increases your risk of lung cancer by *Y *times" *and *"Living within *X *miles of a mobile phone pylon for *N *years increases your risk of brain cancer by *Y *times"*, for any given range of *X *and *Y *values. Lipton and Ødegaard thus argue for better stories, better accounts of study designs, data collection and analysis and more detailed discussion of confounding and potential biases. This is, of course, to be welcomed, as any such increase in rigour can only serve to improve accuracy and precision in describing our observations (see Appendix Footnote 2). However, this still leaves us with the problem of which of these observations are actually true (in the objective, ideal sense). At best, these discussions involve a considerable degree of subjectivity, and decisions regarding which associations are really believable and warrant some form of intervention are reached in a manner resembling an informal and rather undemocratic consensus. Even conceptually more appealing approaches such as multiple bias modelling rely on some consensus about the prior distributions assumed for bias corrections. Thus, when asked whether smoking causes lung cancer, an honest epidemiologist is left merely with the following as a viable response: *"I can tell you that smoking two packs a day for *N *years increases your risk of lung cancer by 10 times"*. In fact, this is clearly an oversimplification, and they would actually be better off saying: *"I can tell you with 95 percent certainty that smoking two packs a day for *N *years increases your risk of lung cancer by between *A *and *B *times"*. But even this is not satisfactory, and one of these two would be preferable: *"I can tell you with 95 percent certainty that smoking two packs a day for *N *years increases your risk of lung cancer by between *A *and *B *times, assuming that there is no systematic error in my observations" *or *"I can tell you with 95 percent certainty that smoking two packs a day for *N *years increases your risk of lung cancer by between *A *and *B *times, and I have tried to correct for biases *C, D *and *E *using prior distributions *S, T *and *U, *which I believe (though I cannot be certain) are rational and exhaustive"*. By this time, the enquirer will probably have regretted asking the question in the first place and, while puffing away at their cigarette, poured themselves a stiff whisky too.

The problem is that to describe such processes accurately requires the use of very unwieldy language that is not only linguistically unappealing, but also unfamiliar to most of those who would have an interest in whether smoking causes lung cancer. This is well known to any student of introductory statistics who has grappled with statements such as *"There is insufficient evidence to reject the null hypothesis"*. All the goodwill in the world and desire for transparency will not change this fact. It is rather ironic that the field that is best placed to identify adverse effects on people's health is probably the worst-suited for communicating them. This is hardly surprising, for the perspective from which we deliver such statements differs markedly from the perspective of those receiving them. Thus, when the honest epidemiologist asserts that *"I can tell you that smoking two packs a day for *N *years increases your risk of lung cancer by 10 times"*, they are reporting an association obtained within a very specific context that has to do with the way in which their study was designed, the individuals selected to participate in the study, how data were collected and analyzed, issues of bias and confounding, and our knowledge of statistical concepts. When confronted with such a statement, however, an individual might wish to raise some more pointed questions, such as, for example, *"How likely is it that this statement is true for me"*. The honest epidemiologist now has no recourse, for this is not a question that they can answer. We can, of course, say that for some individuals the probability may be exactly 0 and that for others it may be exactly 1 (see Appendix Footnote 3). But here is the crux: while Lipton and Ødegaard favour such statements because they are more accurate, this is only the case within a very specific context, that of epidemiologists conducting the science of epidemiology. To the end user of such information, however, it is this sort of statement that can be misleading, because there is no way in which we can say that it is true for any individual, at least not without calling on some abstract idea of an "average" individual. Yet while epidemiologists like to elevate the term "cause" to a philosophical and mythical realm, as used in everyday life, it has many appealing linguistic qualities. If I were to walk ten minutes away from my office (to get away from the concentration of epidemiologists), randomly stop people on the street and ask them if they think smoking "causes" lung cancer, I expect that most of them would say yes. I also expect that most would interpret the term "cause" in a probabilistic manner, recognizing that not everybody who smokes develops lung cancer, and that not everybody who develops lung cancer is a smoker. I imagine that some will even recognize that among smokers who develop lung cancer, it is not necessarily true to say that it was smoking that led to their illness. The common use of the word "cause" thus encapsulates all the oft-recited qualities of partiality, necessity and sufficiency, while imparting at least some qualitative notions of statistical uncertainty.

By maintaining abstract ideals of objective and unattainable causes, we do a great disservice to our field and those whose interests we aim to protect. The idea of the honest (if not necessarily objective) epidemiologist entrusting others with their carefully observed and qualified associational statements is, to my mind, not entirely satisfactory, because those who can most benefit from that information are not necessarily well equipped to interpret it. The statement *"*X *causes *Y" imparts a sense of conviction – that considering all the available observations, with all their qualifications, our most reasonable interpretation is that, in a real if not necessarily ideal sense, *X *does indeed cause *Y*, to an extent that something should be done about it. To shy behind associational statements, though accurate and transparent, is to shun this sense of social responsibility – that we, as gatherers, processers and interpreters of data, should be compelled to act based upon our observations, and not merely leave them to the interpretations of others whose judgement is perhaps no better than our own. The problem still remains of when and by what criteria we consider evidence to be sufficient to warrant action, and this will be an on-going debate, one that will additionally involve politics, economics and social values, and in which epidemiologists should undoubtedly be increasingly involved. Whether we call them "causes" or "roses" is, to a large extent, a moot point. There is, however, no word in the English language, or in any of the languages with which I am familiar, to describe an association for which there is sufficient evidence to warrant some form of intervention. Perhaps epidemiologists should invent one. For those bourgeoning students of introductory statistics, however, it will be depressing to hear that we can never really know that we are right in ascribing causal associations. Despite our best intentions and qualifications (and at the risk of sounding like a refutationist), at best all we can say is that so far we haven't been not right. But in the greater scheme of things, epidemiologists can only ever be wrong.

## Competing interests

I declare that I have no competing interests (other than being an epidemiologist).

## Appendix

### Footnote 1

Lipton and Ødegaard use the term "real" to describe metaphysical, objective truth. I use the term here throughout the text to describe our everyday world, referring to objective truths as "ideals".

### Footnote 2

I refer to "accuracy and precision in describing our observations" in the sense of our statements about associations being more accurate and precise descriptions of our observations, rather than in the sense of them being necessarily true (in the objective, ideal sense).

### Footnote 3

So as not to exclude the stochasticists among us, I should also add here that for some individuals, perhaps even for most individuals, the probability might be somewhere between zero and one.
